# Strain-engineered growth of two-dimensional materials

**DOI:** 10.1038/s41467-017-00516-5

**Published:** 2017-09-20

**Authors:** Geun Ho Ahn, Matin Amani, Haider Rasool, Der-Hsien Lien, James P. Mastandrea, Joel W. Ager III, Madan Dubey, Daryl C. Chrzan, Andrew M. Minor, Ali Javey

**Affiliations:** 1 Electrical Engineering and Computer Sciences, University of California at Berkeley, Berkeley, CA 94720 USA; 2 Materials Sciences Division, Lawrence Berkeley National Laboratory, Berkeley, CA 94720 USA; 3 Department of Materials Science, University of California at Berkeley, Berkeley, CA 94720 USA; 4 National Center for Electron Microscopy, Molecular Foundry, Lawrence Berkeley National Laboratory, Berkeley, CA 94720 USA; 5 US Army Research Laboratory, 2800 Powder Mill Road, Adelphi, MD 20783 USA

## Abstract

The application of strain to semiconductors allows for controlled modification of their band structure. This principle is employed for the manufacturing of devices ranging from high-performance transistors to solid-state lasers. Traditionally, strain is typically achieved via growth on lattice-mismatched substrates. For two-dimensional (2D) semiconductors, this is not feasible as they typically do not interact epitaxially with the substrate. Here, we demonstrate controlled strain engineering of 2D semiconductors during synthesis by utilizing the thermal coefficient of expansion mismatch between the substrate and semiconductor. Using WSe_2_ as a model system, we demonstrate stable built-in strains ranging from 1% tensile to 0.2% compressive on substrates with different thermal coefficient of expansion. Consequently, we observe a dramatic modulation of the band structure, manifested by a strain-driven indirect-to-direct bandgap transition and brightening of the dark exciton in bilayer and monolayer WSe_2_, respectively. The growth method developed here should enable flexibility in design of more sophisticated devices based on 2D materials.

## Abstract (web summary)

Strain engineering is an essential tool for modifying local electronic properties in silicon-based electronics. Here, Ahn et al. demonstrate control of biaxial strain in two-dimensional materials based on the growth substrate, enabling more complex low-dimensional electronics.

## Introduction

Two-dimensional (2D) transition metal dichalcogenides (TMDCs) have been the subject of focused research owing to their potential applications in optoelectronics and sub 10 nm transistors^1, 2^. The primary attraction of TMDCs such as MoS_2_ and WSe_2_ for both applications is their naturally terminated surface, which allows them to be scaled down to the atomic limit without the concern of surface dangling bonds. Furthermore, in many 2D materials, a number of desirable properties emerge at the monolayer limit, the most notable of which being the presence of a direct bandgap. Many studies based on mechanical bending of exfoliated 2D TMDCs have been conducted on flexible substrates, and they have shown that the application of strain can tune the properties of this new class of materials^3–6^. For example, it has been demonstrated that in multilayer WSe_2_, particularly in nominally indirect-gap bilayer WSe_2_, application of tensile strain can result in a transition from an indirect-to-direct bandgap^7^. However, studies on the effects of strain in TMDCs have been limited to mechanical bending of samples, and there has yet to be a technique, which can directly realize built-in tensile and compressive strains.

Traditionally, strain engineering of semiconductors has been leveraged to tune the electronic band structure of high-performance devices, the most notable of which being to reduce intervalley scattering, increase mobility in Si transistors, and reduce the hole effective mass in III–V semiconductor lasers^8, 9^. Growth on epitaxial substrates with a controlled lattice constant mismatch has typically been utilized to establish built-in strain in three-dimensional semiconductors^10^. However, due to the relatively weak interaction between 2D materials and substrates, this established method of strain engineering is likely not applicable for the strain-engineered growth of TMDCs. Therefore, a route toward development of large-area strained TMDCs on a practical substrate is highly desirable. Significant research efforts have been made to realize large area TMDCs, with the majority of efforts focusing on chemical vapor deposition (CVD)^11–13^. Interestingly, a number of studies employing CVD growth of 2D materials have reported apparent strain in the synthesized samples^14, 15^. However, growth of high-quality 2D materials with controllable built-in strain has not been realized. Hence, properties that are difficult to probe via mechanical bending experiments, such as low-temperature optical measurements and electrical performance, have yet to be explored.

In this work, we demonstrate strain engineering of 2D materials directly via CVD growth while simultaneously maintaining high material quality, by utilizing the thermal coefficient of expansion (TCE) mismatch between the TMDC and the growth substrate. Electron diffraction of strained monolayers grown directly onto transmission electron microscopy (TEM) windows is utilized to unambiguously quantify strain. Using WSe_2_ as a model system, we show that it is possible to obtain both tensile (of ~ 1%) and compressive (0.2%) strained 2D semiconductors over large areas on rigid substrates. Furthermore, in the WSe_2_ model system we show indirect-to-direct optical transition in tensile strained WSe_2_ bilayers as well as removal of dark exciton quenching in WSe_2_ monolayers.

## Results

### TCE-mismatch induced strain

**Fig. 1:**
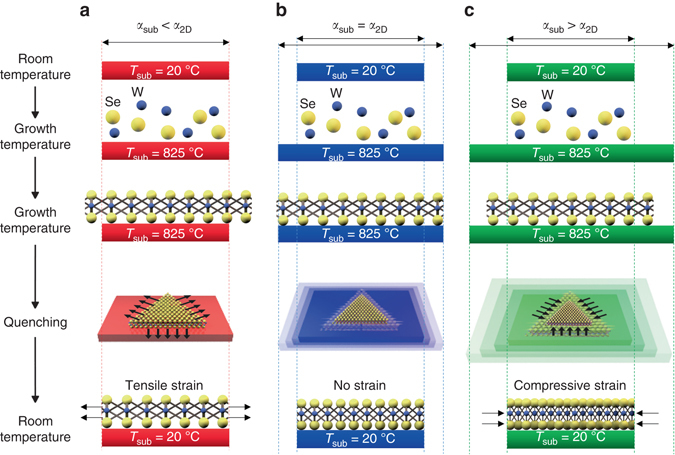
Strain-engineered growth of WSe_2_ using TCE-mismatch. Schematic of the process through which strain is realized during the growth of 2D materials via mismatch in substrate and TMDC thermal expansion coefficient. **a** Tensile strain is achieved when the TCE of the substrate is less than that of the 2D material, **b** relaxed samples are achieved when the TCE of the substrate and 2D material match, and **c** compressive strain is achieved when the TCE of the substrate is greater than that of the 2D material

Owing to the high growth temperatures used to synthesize TMDCs, the TCE mismatch between the substrate and the 2D semiconductor can be utilized to control the strain in the synthesized 2D material, as shown schematically in Fig. 1. The theoretical upper limit for the strain, assuming no relaxation, can be calculated using the difference in lattice constant of the substrate and 2D semiconductor at room temperature and synthesis temperature:

1 $$\varepsilon \left( {{T_{\rm{g}}}} \right) = \frac{{{a_{2{\rm{D}}}}\left( {{T_{\rm{g}}}} \right) - {a_{2{\rm{D}}}}\left( {25\,^\circ {\rm{C}}} \right)}}{{{a_{2{\rm{D}}}}\left( {25\,^\circ {\rm{C}}} \right)}} - \frac{{{a_{{\rm{Sub}}}}\left( {{T_{\rm{g}}}} \right) - {a_{{\rm{Sub}}}}\left( {25\,^\circ {\rm{C}}} \right)}}{{{a_{{\rm{Sub}}}}\left( {25\,^\circ {\rm{C}}} \right)}}$$

where *a*_2D_ is the in-plane lattice constant for the 2D material being grown, *a*_Sub_ is the in-plane lattice constant of the substrate, and *T*_g_ is the growth temperature. More generally, this can be calculated using the temperature dependent thermal expansion coefficient for the 2D material (*α*_2D_) and substrate (*α*_Sub_) according to:

2 $$\varepsilon \left( {{T_{\rm{g}}}} \right) = \mathop {\int}\limits_{25\,^\circ {\rm{C}}}^{{T_{\rm{g}}}} {{\alpha _{2{\rm{D}}}}\left( T \right){\rm{d}}T} - \mathop {\int}\limits_{25\,^\circ {\rm{C}}}^{{T_{\rm{g}}}} {{\alpha _{{\rm{Sub}}}}\left( T \right){\rm{d}}T}$$

To retain the strain generated from TCE-mismatch, the bonding between the 2D layer and the substrate must be sufficiently strong and corrugated to maintain the nonslip condition that transmits strain to the layer. Although one expects strains of this magnitude to arise during epitaxial growth, it is difficult to see how a similar strain can arise for films bound by van der Waals forces to an amorphous substrate. Experimentally, the corrugation in the film must be large enough to retain the strain upon cooling. To estimate the magnitude of the corrugation necessary to sustain the strain, we analyzed a 2D Frenkel–Kontorova model^16^, shown in Supplementary Note 1 and Supplementary Fig. 1. This model suggests that atomic binding energy variations of as little as 14 meV over the range of the unit cell are capable of retaining strains of the magnitude of those observed experimentally. This variation is ~ 1% of a typical covalent bond strength, and very near to the typical strength of a van der Waals bond. Though a more detailed theory needs to be developed, especially to understand the surface chemistry immediately prior to growth to determine the nature of the bonding, this simple analysis suggests that thermal-expansion mismatch can strain a film, even in the case of weak binding between the film and the substrate. It should be noted that in a previous study, local heating of MoS_2_ using a laser was used to generate strains of up to 0.2% arising from the TCE-mismatch of MoS_2_ with the substrate^17^. Although this method does not generate built-in strains (strain is released once the laser is turned off), it suggests that the MoS_2_-substrate bonding is strong enough to enable TCE-mismatched induced strain engineering^17^.

To demonstrate strain-engineered CVD growth of WSe_2_, four substrates were chosen, which have a range of TCEs. Fused silica and aluminum nitride (AlN) have a TCE of 0.55 and 5.5 ppm, respectively, much smaller than that of WSe_2_ (9.5 ± 3.2 ppm), and would be expected to induce tensile strain^3, 18–22^. In contrast, sapphire has a TCE closely matched to WSe_2_ and would be expected to produce relaxed samples and strontium titanate (STO) with a TCE of 12 ppm should yield compressively strained samples^20, 23, 24^. The sample was rapidly quenched after growth to limit relaxation in the WSe_2_. It is important to note that the rapid quenching process was found to be important in maintaining the presence of strain, and delay of the quenching step can result in partial relaxation of the 2D material.

### Characterization strain and material quality

**Fig. 2:**
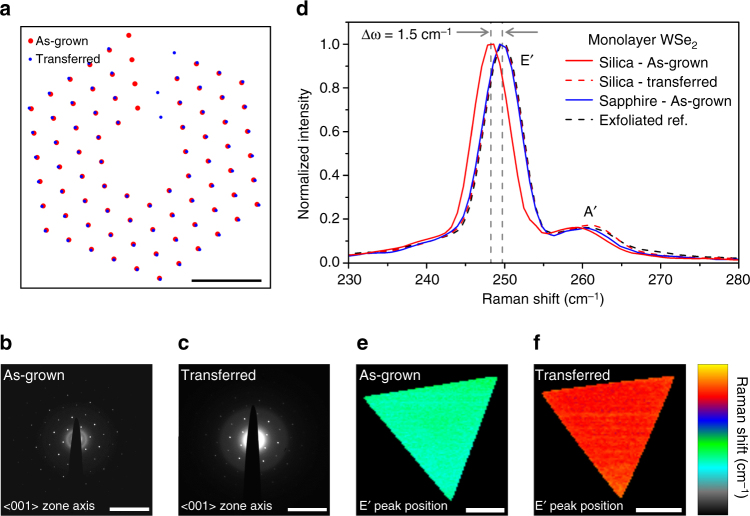
Transmission electron microscopy images and Raman spectroscopy of monolayer WSe_2_. **a** Overlay of diffraction spots for as-grown and transferred monolayer WSe_2_, showing a 1.39 ± 0.28% strain in samples grown directly on the TEM membrane. *Scale bar* is 10 nm^−1^. **b** Electron diffraction patterns of a tensile strained WSe_2_ monolayer grown directly on an 8 nm SiO_2_ TEM membrane and **c** a WSe_2_ monolayer transferred to the same thickness membrane. *Scale bar* is 10 nm^−1^. **d** Raman spectra of WSe_2_ monolayers grown on various substrates. **e**, **f** Raman mapping of E’ peak position for a (**e**) tensile strained WSe_2_ monolayer as-grown on fused silica and a (**f**) WSe_2_ monolayer transferred to release strain; data are plotted using the same false color scheme. *Scale bar* is 20 μm, data range is 250.5 to 247.0 cm^-1﻿^

To characterize the strain present in the as-grown material, multiple characterization methods were utilized. First, electron diffraction was performed on WSe_2_ as-grown on SiO_2_ TEM grids and WSe_2_ transferred to SiO_2_ TEM grids. The resulting diffraction patterns are shown in Figs. 2a–c. In all TEM diffraction measurements on WSe_2_, the camera length and lens aberrations of the imaging systems were calibrated using polycrystalline aluminum and single crystal aluminum calibration samples (Ted Pella), and the microscope lens settings were left constant for all subsequent measurements. Corresponding images and photoluminescence (PL) spectra of the samples in Figs. 2b, c are shown in Supplementary Fig. 2. By comparing the lattice constant extracted from the diffraction patterns of strained (as-grown) and unstrained (transferred) WSe_2_, and utilizing the center of mass for 75 diffraction spots from each pattern, we calculate that a 1.39 ± 0.28% tensile strain is present in the as-grown sample. The calculated error represents the standard deviation in lattice constant value over 75 measured diffraction spots. Raman spectrum shown in Fig. 2d reveals a shift of the E’ in-plane Raman mode of 1.5 ± 0.2 cm^−1^ between as-grown and transferred WSe_2_. We note that the strain measured here is biaxial, not uniaxial, in contrast to mechanical bending studies and thus we do not observe splitting of the E′ mode. Therefore, the theoretically calculated value for the Grüneisen parameter (﻿*χ*﻿) of WSe_2_ is used to calculate the expected peak shift according to: $\omega \left( \varepsilon \right) = {\omega _0} + {\rm{\chi}}\varepsilon$, which is consistent with the approximately 1% tensile strain measured via diffraction^25–27^. It should also be noted that after transfer the E’ mode shows no shift relative to samples prepared either by exfoliation or as-grown on sapphire. High resolution Raman mapping was performed and is shown in Figs. 2e, f for WSe_2_ crystals as-grown on fused silica and transferred to a new fused silica substrate, respectively. We observe a uniform Raman shift throughout the full WSe_2_ domain for both samples indicating that the strain is uniformly distributed within the sample on the scale of the Raman spot size. In addition, PL and atomic force microscopy images of as-grown WSe_2_ on fused silica are shown in Supplementary Fig. 3.

**Fig. 3:**
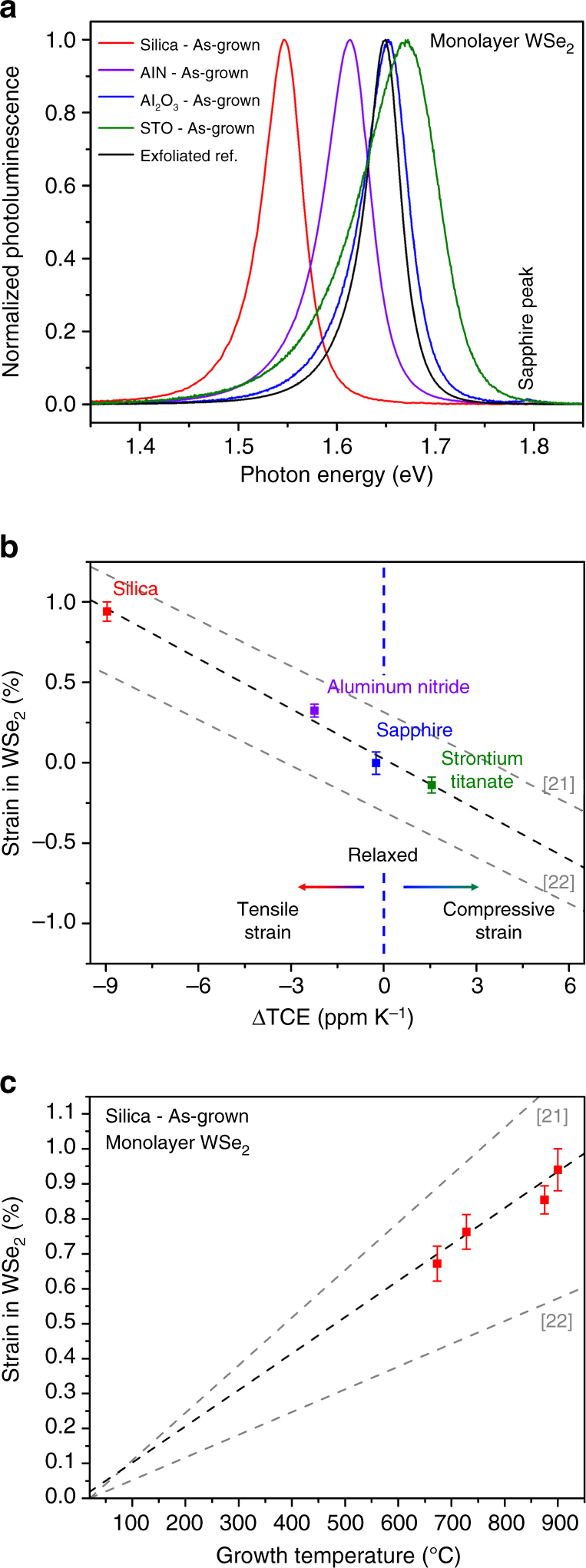
Strain engineering of 2D materials. **a** PL spectra for monolayer WSe_2_ as-grown on substrates with a range of thermal coefficient of expansion mismatches. **b** Estimated strain that can be achieved in 2D materials based on the mismatch between the substrate and TMDC thermal coefficient of expansion; data points show measured strain for substrates with a range of thermal expansion coefficient mismatch; *dashed gray lines* are based on TCE values for WSe_2_ obtained from refs ^21, 22^, *dashed black line* shows fit to experimental data. **c** Estimated strain realized in WSe_2_ as-grown on fused silica at various substrate temperatures; *dashed gray lines* are based on TCE values for WSe_2_ obtained from refs ^21, 22^, *dashed black line* shows fit to experimental data. Error bars indicate standard deviation of strain, measured over fifteen samples

PL measurements were employed to measure the bandgap as a function of strain. Figure 3a shows PL spectra of samples grown on all substrates investigated in this work as well as an exfoliated reference. There is a large spectral shift of 120 meV between samples grown on fused silica with near zero TCE and STO, which has the highest TCE of all substrates investigated in this work. Figure 3b shows the estimated strain from the PL peak shift as a function of predicted strain in WSe_2_, vs. the lattice mismatch between the substrate and TMDC lattice constant. Strain values are determined from the experimentally measured PL peak position (measurement error was calculated using the standard deviation of strain measured from fifteen different crystals) and theoretically calculated bandgap as a function of strain extracted from refs ^3, 27^. It is important to note that we utilized the difference in peak position relative to an unstrained sample to eliminate effects from absolute bandgap error relative to theoretical calculations. We performed an analysis of the expected strain due to lattice parameter misfit (Supplementary Table 1 and Supplementary Note 2) to rule out the influence of epitaxial strain between the substrate and WSe_2_. From this calculation, the expected strain from the lattice mismatch shows the opposite trend as compared with what we observe experimentally. We find that our experimentally realized strain per ΔTCE of −0.10 ± 0.01% per ppm K^−1^ is in excellent agreement with the theoretical value of −0.09% per ppm K^−1^. To verify that the substrate does not affect the PL peak shape or position of monolayer WSe_2_, samples were transferred to the four substrates used in this study (Supplementary Fig. 4) and no shift of the PL peak was observed. The impact of growth temperature to tune the strain was also studied using fused silica as the substrate. Figure 3c shows the tensile strain in monolayer WSe_2_ grown at different temperatures, estimated using PL peak position. We are able to tune the strain from 0.94 ± 0.06% to 0.67 ± 0.05% as the growth temperature is changed from 900 to 673 °C. PL spectra corresponding to Fig. 3c is shown in Supplementary Fig. 5.

### Tensile strain engineering of mono- and bilayer WSe_2_

**Fig. 4:**
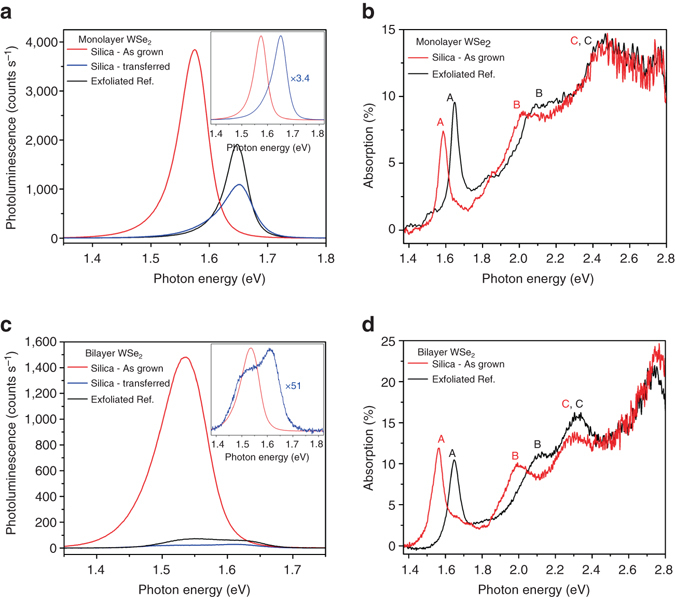
Photoluminescence of strain-engineered monolayer and bilayer WSe_2_. **a** Photoluminescence spectra of as-grown and transferred monolayer WSe_2_ as well as exfoliated reference. Inset shows normalized spectra. **b** Absorption spectra of as-grown and exfoliated monolayer WSe_2_. **c** Photoluminescence spectra of as-grown and transferred bilayer WSe_2_ as well as exfoliated reference; inset shows normalized PL spectra of as-grown and transferred bilayer WSe_2_. **d** Absorption spectra of as-grown and exfoliated bilayer WSe_2_

Calibrated PL measurements were used to provide a gauge of material quality. We find that strained WSe_2_ monolayers as-grown on fused silica show comparable PL intensity to exfoliated WSe_2_, demonstrating that strain engineering of 2D materials can be achieved without compromising material performance. Specifically, as exfoliated samples show a typical quantum yield (QY) of 2.3 ± 0.4%, while samples grown on fused silica show a typical QY of 1.9 ± 0.6%. Upon transfer, the strain within the as-grown material is released and its emission peak shifts to 1.65 eV, closely matching that of exfoliated samples, as shown in Fig. 4a. In addition to emission, the absorption spectrum of the as-grown monolayer was measured and is shown in Fig. 4b. The absorption spectra clearly shows that the A and B exciton resonances are red-shifted with the presence of tensile strain, and are consistent with the observed shift in the emission. Interestingly, the absorption of the C exciton peak shows no shift between the as-grown and exfoliated samples, which is consistent with previously reported mechanical bending experiments on exfoliated monolayers, although it should be noted that in these experiments the strain was uniaxial^28^.

More interestingly, it has been previously demonstrated using bending experiments that under the application of a uniaxial strain of ~ 1.5%, bilayer WSe_2_ undergoes an indirect-to-direct bandgap transition^7^. This effect is caused by an increase in the energy level of the indirect valley at the Σ point as well as a corresponding decrease in the energy level of the direct valley at the K point^7^. While it is important to note these results are obtained under uniaxial strain, similar trends between uniaxial and biaxial strain have been observed in theoretical studies on other 2D material systems^29^. Figure 4c depicts the PL spectra of as-grown, exfoliated and transferred bilayer WSe_2_. In the as-grown strained bilayer, we observe a single sharp PL peak, as oppose to a broader spectrum corresponding to emission from both the indirect and direct bandgaps observed in the unstrained exfoliated and transferred samples. Moreover, the intensity of the PL is reduced by approximately 50 times upon transfer and resultant release of tensile strain as shown in Fig. 4c. This phenomenon can possibly be attributed to the optical transition from indirect-to-direct in bilayer WSe_2_ in the presence of tensile strain. This is in good agreement with previous reports on the indirect-to-direct optical transition of WSe_2_ through bending experiments, although these previous studies utilized uniaxial strain^7^. The normalized PL spectra for bilayer WSe_2_ grown on fused silica after transfer, as well as unstrained samples grown on sapphire and prepared by exfoliation, are shown in Supplementary Fig. 6 and show a very similar spectral shape. Absorption of WSe_2_ bilayer samples was also measured and is shown in Fig. 4d.

**Fig. 5:**
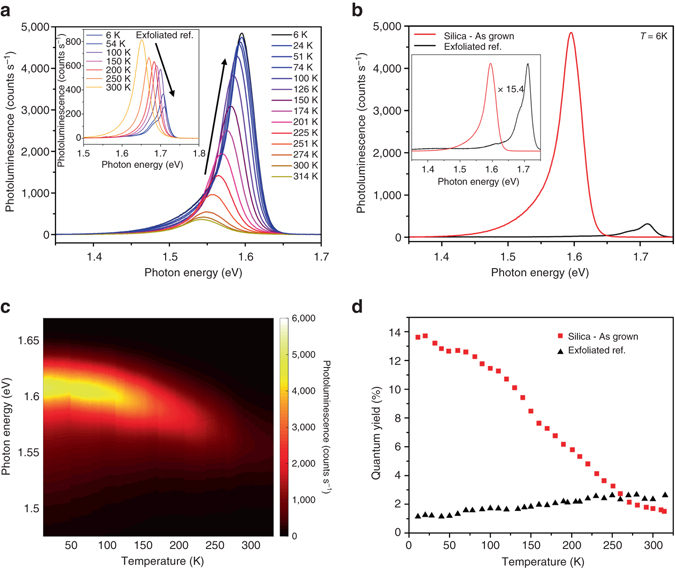
Low-temperature photoluminescence of strain-engineered monolayer WSe_2_. **a** Temperature dependent photoluminescence spectra of as-grown monolayer WSe_2_ (tensile strained), inset shows the same measurement on an exfoliated monolayer WSe_2_ sample. **b** Photoluminescence spectra of as-grown (tensile strained) WSe_2_ and exfoliated WSe_2_ measured at 6 K, inset shows normalized spectra. **c** Two dimensional plots of photoluminescence spectra and intensity of as-grown (tensile strained) monolayer WSe_2_. **d** Temperature dependence of the PL QY for as-grown (tensile strained), and exfoliated WSe_2_ as a function of temperature

To further characterize WSe_2_ monolayers, we performed low-temperature PL measurements. Numerous studies have experimentally observed that the PL of monolayer WSe_2_ is quenched at reduced temperatures. This behavior is unusual for a direct bandgap semiconductor and has been attributed to the formation of dark excitons with an energy level ~ 30 meV below the bright state^30, 31^. It has been hypothesized that at low temperatures the bright exciton is allowed to relax to the lower energy level resulting in a reduction of the PL QY. Conversely, both theoretical and experimental studies have suggested that unstrained monolayer WSe_2_ is an indirect-gap semiconductor, with a small (< 3kT) energy difference between the indirect and direct gaps^32, 33^. Upon cooling of exfoliated monolayer WSe_2_ samples, we observe results similar to what has been previously reported as shown in the inset of Fig. 5a, which is a reduction in PL emission at low temperatures. However, for the case of tensile strained monolayer WSe_2_ as-grown on fused silica we observe the opposite trend (Fig. 5). As the temperature is reduced from 300 to 6 K the PL intensity increases by over an order of magnitude, resulting in a final PL QY of 14% as shown in Fig. 5d. The nature of the physical mechanism for the dramatic difference in the temperature-dependent behavior can be directly attributed to band structure modification due to the presence of strain. However, it remains unclear if this is due to an indirect-to-direct bandgap transition or an increase in the energy level of the dark state.

## Discussion

In summary, we have demonstrated controlled and stable strain engineering of 2D materials by growth, realized through TCE mismatch between the substrate and 2D material. The demonstrated strain engineering allows for dramatic modulation of the physical properties of 2D materials. Using WSe_2_ as a model system, we have achieved a strain induced indirect-to-direct optical transition in directly grown WSe_2_ bilayer, resulting in an amplification of the PL intensity by over fifty times. We have also shown tunable PL emission of WSe_2_ monolayer, whereas maintaining its high performance. Notably, in the monolayer case under tensile strain, we observed increasing luminescence at reduced temperatures, in stark contrast with what has been reported in the unstrained exfoliated material, suggesting that strain can be utilized to brighten the dark exciton. This practical route for the direct realization of strain engineering in 2D materials can allow for the characterization of device properties as a function of strain as well as enable optical measurements that are not accessible to samples strained by physical bending or stretching. Our work depicts that biaxial strains of 1% tensile to 0.2% compressive can be obtained by using readily achievable differences in the thermal expansion coefficients by choosing an appropriate growth substrate. By expanding upon this method, it may be possible to obtain uniaxial strain via growth on substrates with anisotropic thermal expansion coefficients or obtain significantly higher strain values via growth on piezoelectric substrates^34, 35^.

## Methods

### Material growth

WSe_2_ was grown on several substrates with varying thermal expansion coefficients using CVD. Specifically, growth was performed on fused silica (amorphous), aluminum nitride (c-plane), Sapphire (c-plane), or strontium titanate (<100>); all substrates were electronic grade and have a root mean square surface roughness of <10 Å. Substrates were first cleaned by sonication in acetone and isopropyl alcohol for 10 min. The growth was carried out using a two-heating-zone furnace (DaePoong Industry, 50602); a schematic of the growth setup is shown in Supplementary Fig. 7. The cleaned substrates were loaded into the downstream zone, and a ceramic boat containing mixture of WO_3_ and potassiu﻿m bromide (KBr) was then placed next to the substrates. KBr was mixed with WO_3_ at a ratio of 1:2, with the KBr acting as a promoter for the growth. This is similar to methods described in ref. ^36^. However, we found that several salts can be used and the choice of promoter has a significant role in determining the ultimate sample morphology (this is further discussed in the Supplementary Methods). It should be noted that regardless of the promoter used, the strain present in the sample was unaffected as shown in Supplementary Fig. 8. A ceramic boat containing 500 mg Se powder was placed in the center of the upstream zone. After loading, the quartz tube was evacuated and Ar was introduced at 60 sccm resulting in a pressure of 2.6 Torr. The temperature of upstream zone was then ramped to 100 °C and the downstream furnace was subsequently ramped to a setpoint of 825 °C. The set point of the upstream zone was adjusted such that the residual heat from the downstream zone results in vaporization of Se. Once the temperature of downstream furnace was stabilized, hydrogen was introduced at a flow rate of 40 sccm (total pressure of 3 Torr). This initiates the growth as the presence of hydrogen is required to decompose and vaporize WO_x_^37^. The growth time used was 15 min. Once the growth is complete, hydrogen flow was stopped, and the furnace was opened to rapidly cool the sample. The influence of the H_2_/Ar ratio, growth time and growth pressure is discussed in the Supplementary Methods and are shown in Supplementary Figs 9–11. Optical microscope images of samples grown on various substrates is shown in Supplementary Fig. 12. Specific growth conditions were tuned to optimize the PL QY of the as-grown samples and to either promote growth of bilayer or monolayer domains (discussed in Supplementary Methods). It should be noted that for growths where the substrate temperature was varied the WO_3_/KBr boat was placed in the center of the first heating zone and the Se boat was placed at the edge of the first heating zone. The substrates were placed in the center of the second heating zone. The position of Se boat was adjusted to control the temperature. Transfer of grown WSe_2_ is done using a HF based wet transfer technique with PMMA as the transfer medium using the specific procedure described in ref. ^11^. The electrical performance of CVD WSe_2_ grown on fused silica and transferred to Si/SiO_2_ is also compared to that of exfoliated samples, and is shown in Supplementary Fig. 13.

### Transmission electron microscopy

WSe_2_ monolayers were directly grown and transferred on 8 nm thick SiO_2_ membranes (Ted Pella) for electron diffraction measurements in order to directly measure the lattice constant of the strained and unstrained samples, respectively. Electron diffraction measurements were performed at the National Center for Electron Microscopy at LBNL using a FEI Titan 60–300 microscope operated at 80 kV with parallel beam illumination.

### Optical characterization

The PL data presented in this work was obtained using a custom built micro-PL instrument described in detail in ref. ^1^. In brief, measurements were performed using the continuous wave 514.5 nm line of an Ar^+^ laser (Lexel 95) and the laser power was adjusted using neutral density filters. The laser was focused on the sample using an 80 × objective lens (NA = 0.9). The PL signal was measured using the same objective lens, passed through a 550 nm dielectric longpass filter, dispersed by a *f* = 340 mm spectrometer with a 150 g mm^−1^ grating, and detected using by an Si CCD camera (Andor iDus BEX2-DD). Prior to each measurement the CCD background was obtained and subsequently subtracted from the PL acquisition. The sensitivity of the instrument as a function of wavelength (instrument function) was determined through measurement of a virtual Lambertian blackbody source under the objective, created by imaging the illumination from a temperature stabilized lamp (ThorLabs SLS201) on a diffuse reflector (Spectralon). The system efficiency was determined by measuring the response of the excitation laser focused onto a diffuse reflector (Spectralon), with the 550 nm longpass filter removed. The measured external quantum efficiency was converted to QY using sample absorption at the pump wavelength and the fraction of light, which is able to escape the sample (1/4*n* ^2^, where *n* is the refractive index of the medium). All measurements shown in this work were performed at an incident power of 1.5 W cm^−2^ (corresponding to a laser power of 200 nW). This laser power is significantly below the onset of biexcitonic recombination in WSe_2_^38^. Low temperature measurements were performed using a long working distance 50 × objective lens in a micro-cryostat (Janis) with an infrasil window. The procedure described previously was used to calculate the sample PL efficiency. PL imaging was performed on the samples to determine their uniformity. Measurements were performed using a florescence microscopy setup using a 470 nm light emitting diode as the excitation source and a CCD detector (Andor Luca). Raman measurements and high-resolution mapping were performed to characterize the strain in the WSe_2_. Raman spectra were obtained using a WITec Alpha 300RA equipped with a piezo electric scanning stage. The sample was excited using the 532 nm line of a frequency-doubled Nd:YAG laser as the excitation source and focused on the sample using a 100 × objective.

### Data availability

The data that support the findings of this study are available from the corresponding author on request.

## Electronic supplementary material

Supplementary Information
