# Supplementary material for: Strain-engineered growth of two-dimensional materials
Source: Nat Commun. 2017 Sep 20;8:608. doi: 10.1038/s41467-017-00516-5 (PMC5606995; doi:10.1038/s41467-017-00516-5)
Supplement: Supplementary file 1 — Supplementary Information [file 41467_2017_516_MOESM1_ESM.pdf]

Supplementary Figures

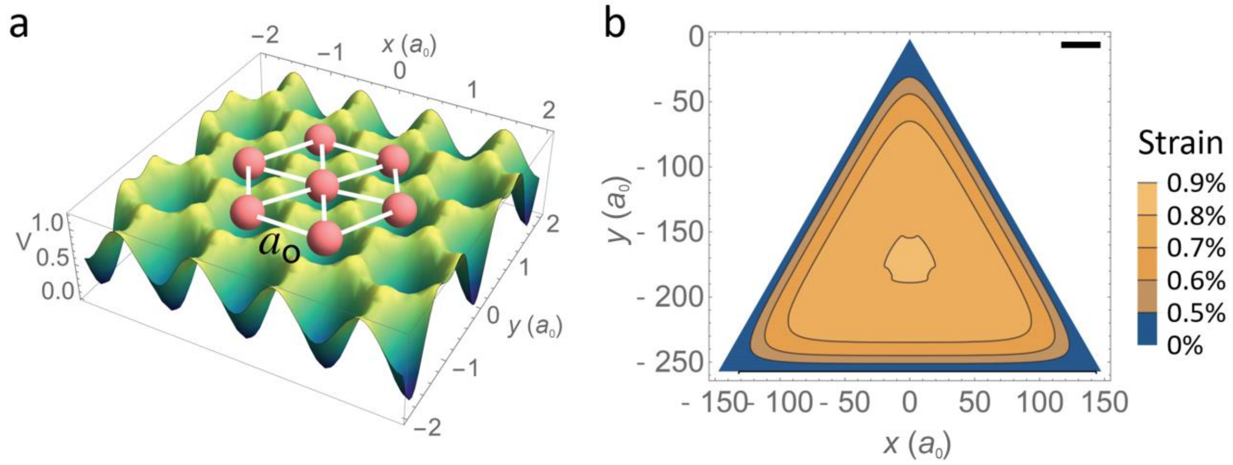

Supplementary Figure 1 | Modeling of binding energy required for retention of TCE mismatch strain. (**a**) Schematic of a network of atoms, shown in pink, connected by springs (indicated by the white lines) with spring constant κ and equilibrium length a_{o}, used to model retention of TCE mismatch strain. The atoms are interacting with a substrate potential measured in terms of $\frac{1}{2}\kappa a_s^2$, where a_{s} is the substrate lattice parameter. The lattice parameter of the periodic potential (a_{o}) is used as the length scale. The model is strictly two-dimensional. (**b**) The strain within the film for $V_{\mathrm{o}} = 5 \times 10^{-4}$ and $a_{\mathrm{o}} = 0.992\ a_{\mathrm{s}}$. Note that the majority of the film is strained over 0.6%, indicating that it retained the high temperature lattice parameter of the substrate. Note also the pattern of relaxation: the edges of the film do relax over a very narrow range. Scale bar is 10 nm and correlates to a choice of $a_{\mathrm{s}} = 3.33$Å.

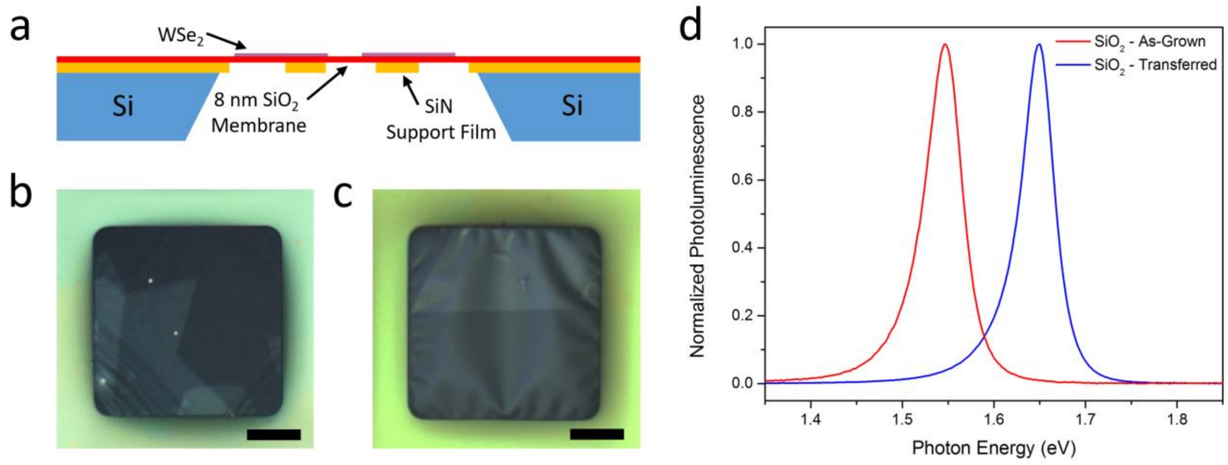

Supplementary Figure 2 | Photoluminescence of monolayer WSe_2 transferred and grown on TEM grid. (**a**) Cross sectional schematic of WSe_2 grown on an 8 nm thick SiO_2 TEM window (TedPella 21532-10). (**b**) Optical micrograph of a monolayer WSe_2 crystal directly grown on a SiO_2 TEM window. Scale bar is 20 µm. (**c**) Optical micrograph of a monolayer WSe_2 crystal transferred on a SiO_2 TEM window. Scale bar is 20 µm. (d) Photoluminescence spectra of the as-grown and transferred WSe_2 flakes, measured using an excitation wavelength of 514.5 nm and incident power density of 1.5 W cm^{-2}; electron diffraction measurements were performed on the samples shown here. Note that PL measurements were taken before TEM.

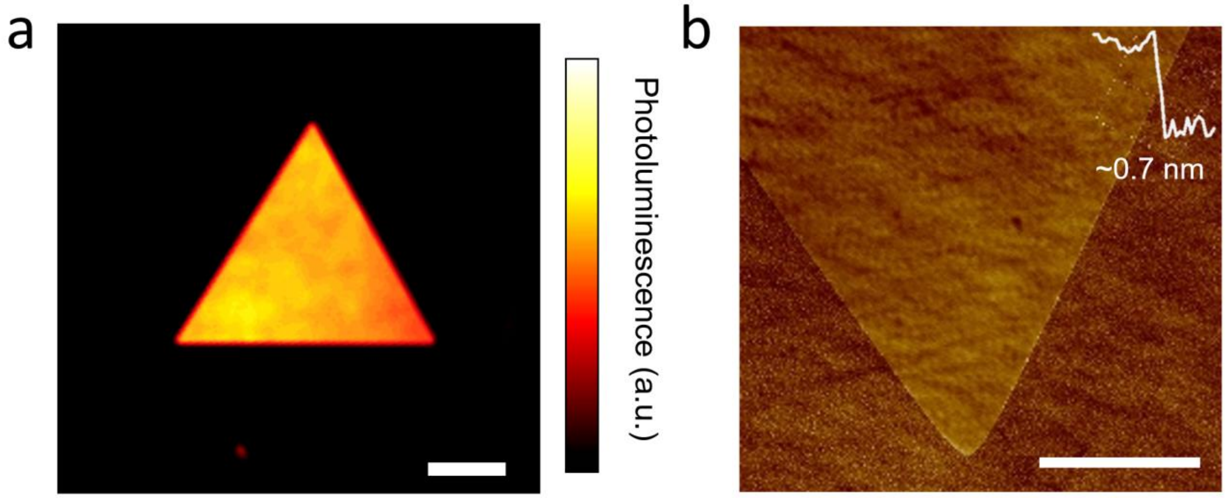

Supplementary Figure 3 | Further characterization of as-grown monolayers. (**a**) Photoluminescence imaging of an as-grown WSe_2 monolayer, excited by a 470 nm LED. Scale bar is 20 μm. (**b**) AFM image of an as-grown WSe_2 monolayer on fused silica, step profile is shown in the inset. Scale bar is 5 μm.

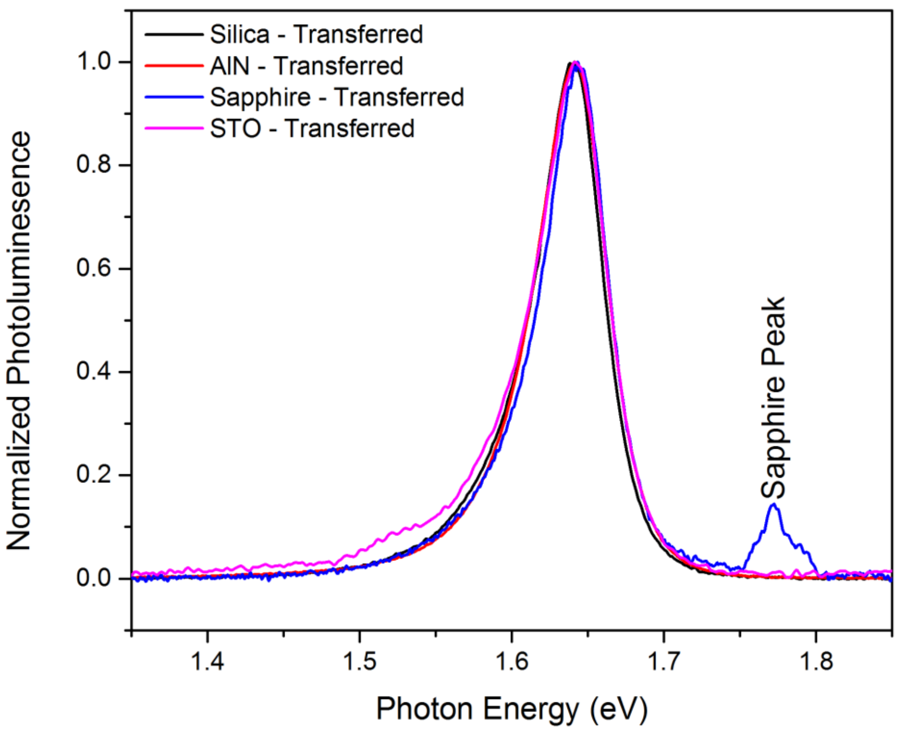

Supplementary Figure 4 | Photoluminescence of monolayer WSe_2 flake transferred to various substrates. Normalized photoluminescence spectra, measured using an excitation wavelength of 514.5 nm and incident power density of 1.5 W cm^{-2}, of the same WSe_2 monolayer sequentially transferred to AlN, sapphire, STO, and fused silica.

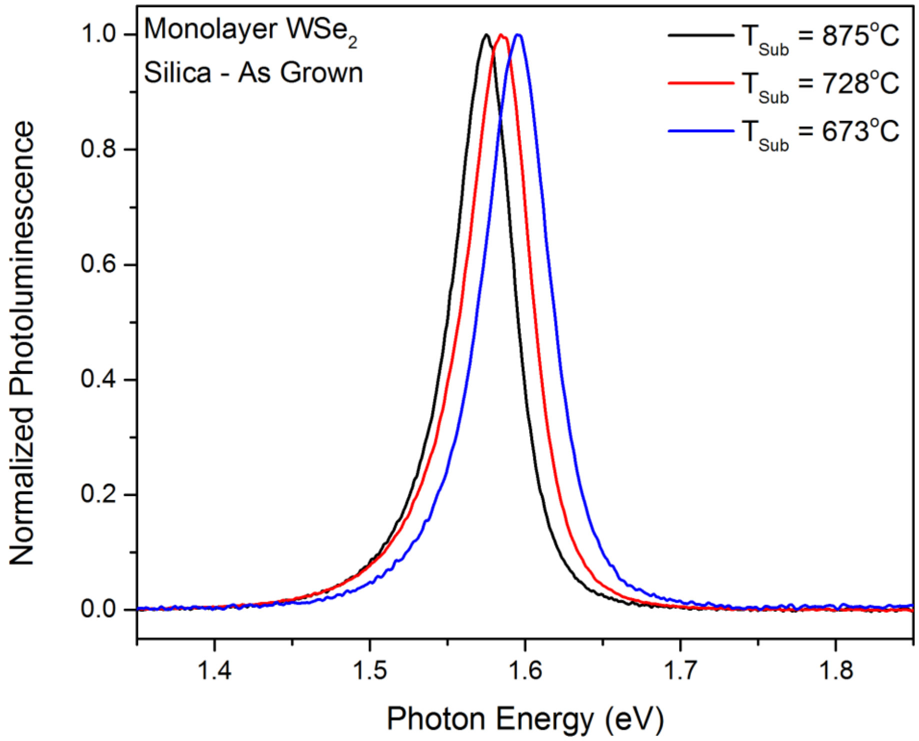

Supplementary Figure 5 | Photoluminescence of monolayer WSe_2 grown as a function of substrate temperature. Normalized photoluminescence spectra, measured using an excitation wavelength of 514.5 nm and incident power density of 1.5 W cm^{-2}, of WSe_2 monolayers as-grown on fused silica with varying substrate temperatures.

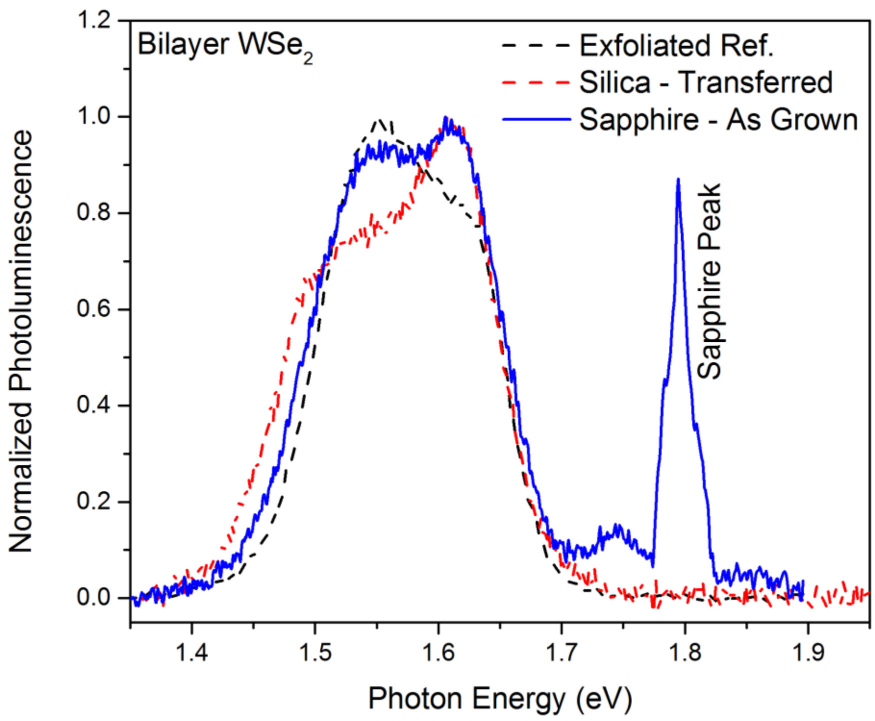

Supplementary Figure 6 | Photoluminescence of relaxed bilayer WSe_2. Normalized photoluminescence spectra, measured using an excitation wavelength of 514.5 nm and incident power density of 1.5 W cm^{-2}, of WSe_2 bilayers prepared by exfoliation, as-grown on sapphire, and transferred from fused silica.

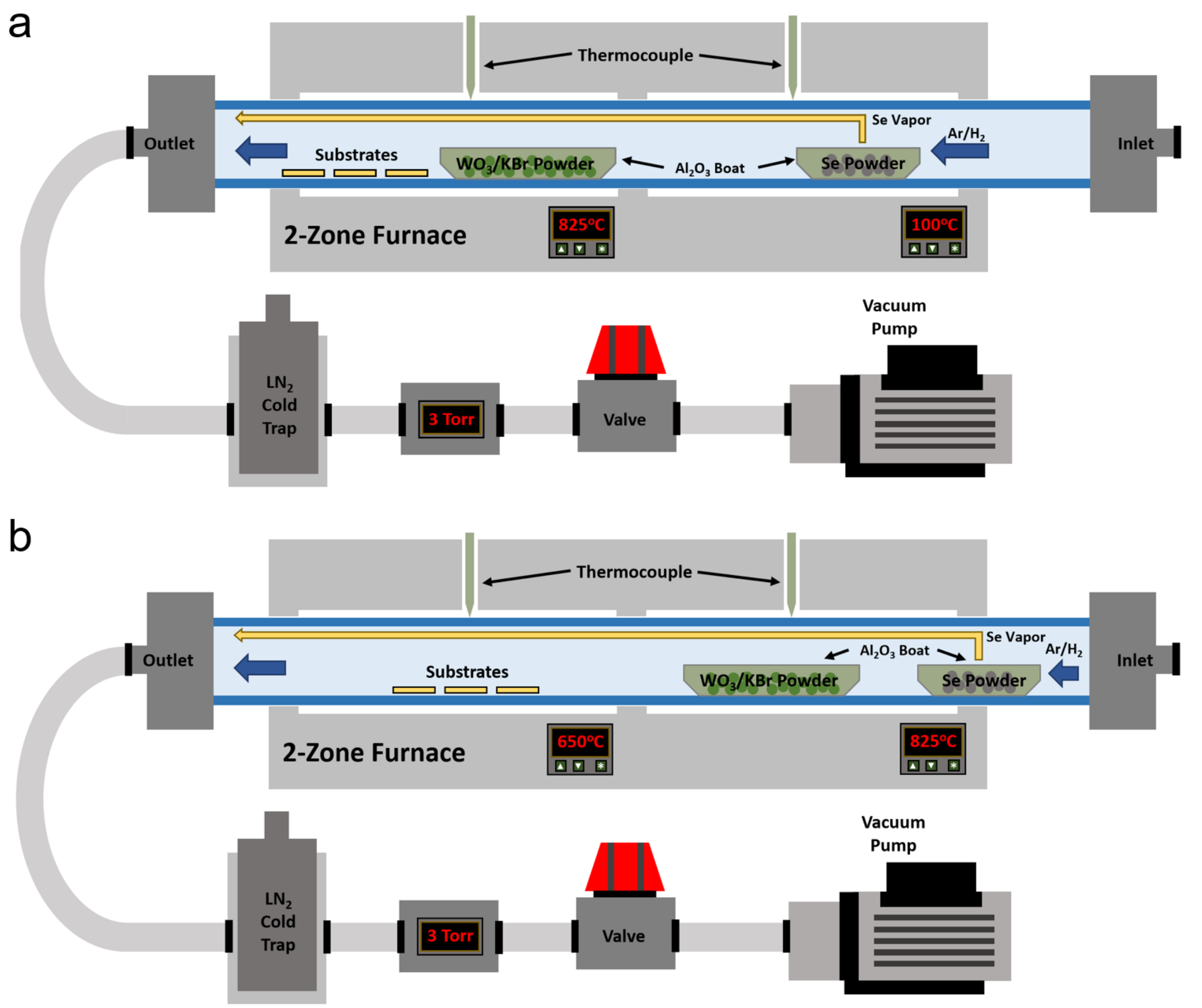

Supplementary Figure 7 | Schematic of growth setup. Illustration of two-zone furnace used for all growths reported in this study. For all growths, except for those where the substrate temperature was varied, the precursor and substrate configuration in panel (**a**) was used. However, for experiments where the substrate temperature was varied (Fig. 3 (**c**)) the configuration in panel (**b**) was used so that the WO_3/KBr boat temperature could be independently controlled.

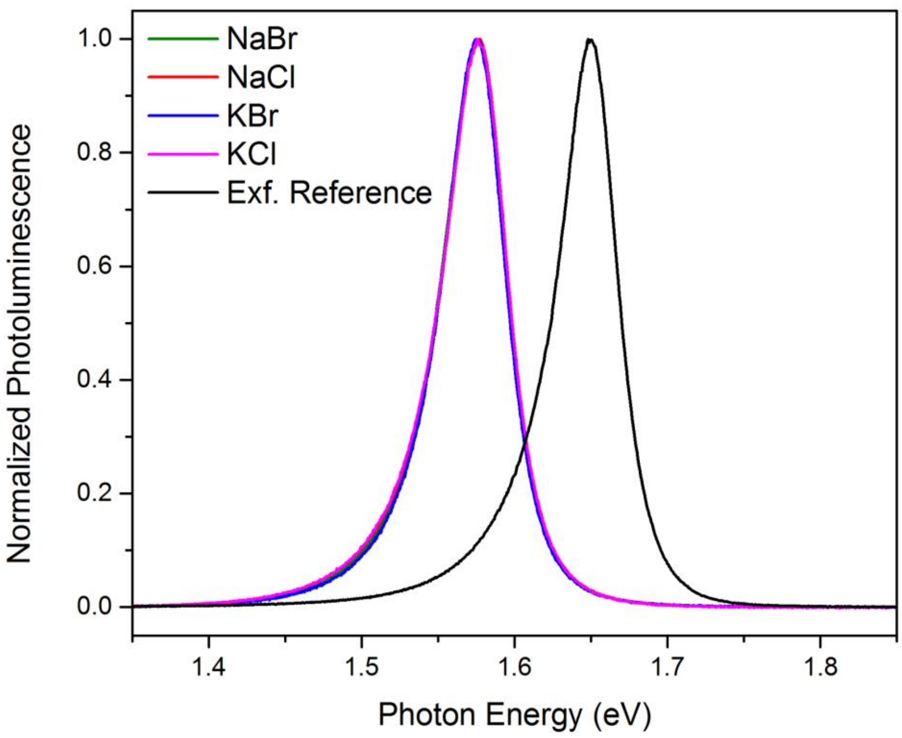

Supplementary Figure 8 | Impact of growth promotor. Normalized photoluminescence spectra, measured using an excitation wavelength of 514.5 nm and incident power density of 1.5 W cm^{-2}, of WSe_2 grown using NaBr, NaCl, KBr, and KCl as the growth promoter as well as an exfoliated reference.

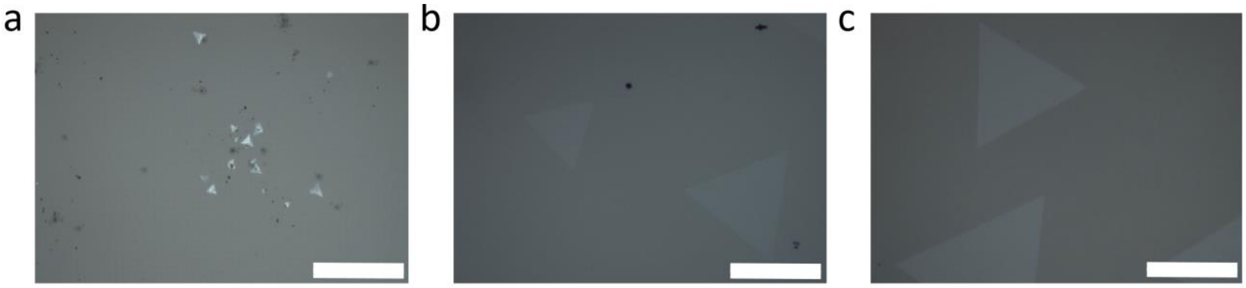

Supplementary Figure 9 | Gas Ratio Dependence of as-grown WSe_2 domains. Typical Optical Images of as-grown WSe_2 grown with hydrogen and argon flow rate of (**a**) 20 sccm/ 60 sccm, (**b**) 40 sccm / 60 sccm, and (**c**) 80 sccm / 60 sccm. Scale bars are 80 μm.

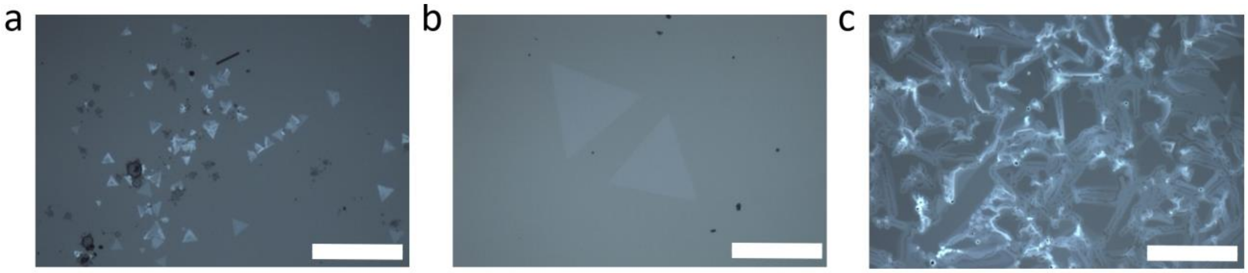

Supplementary Figure 10 | Growth Time Dependence of as-grown WSe_2 domains. Typical Optical Images of as-grown WSe_2 samples grown with growth time of (**a**) 7 minutes, (**b**) 20 minutes, and (**c**) 60 minutes. Scale bars are 80 μm.

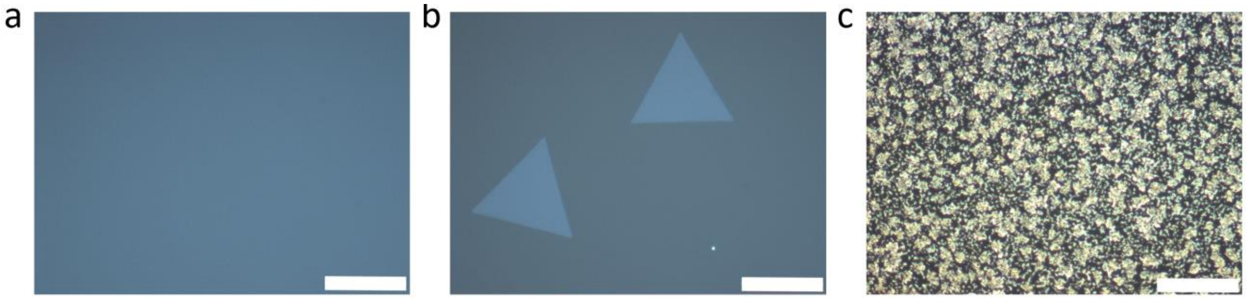

Supplementary Figure 11 | Growth Pressure Dependence of as-grown WSe_2 domains. Typical Optical Images of as-grown WSe_2 grown with pressure of (**a**) 1 Torr (**b**) 3 Torr, and (**c**) 10 Torr. Scale bars are 80 μm.

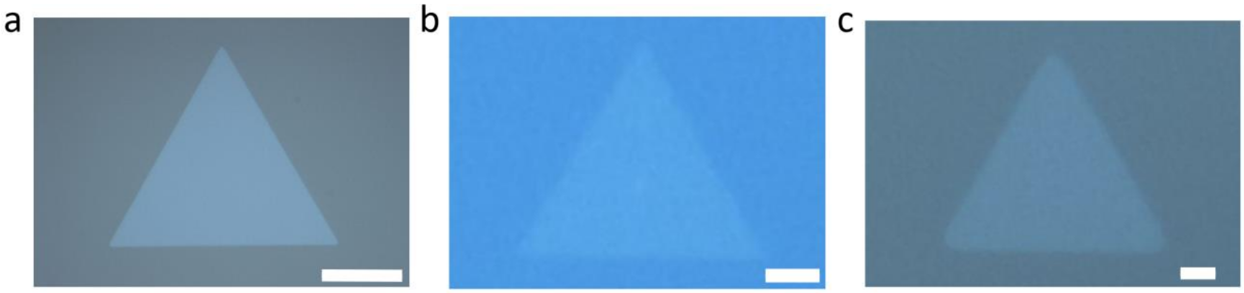

Supplementary Figure 12 | Optical micrographs of as grown monolayers. Optical micrographs of as-grown WSe_2 monolayers on (**a**) fused silica, (**b**) aluminum nitride, (**c**) sapphire. Scale bars are 20 μm, 2 μm, and 2 μm respectively.

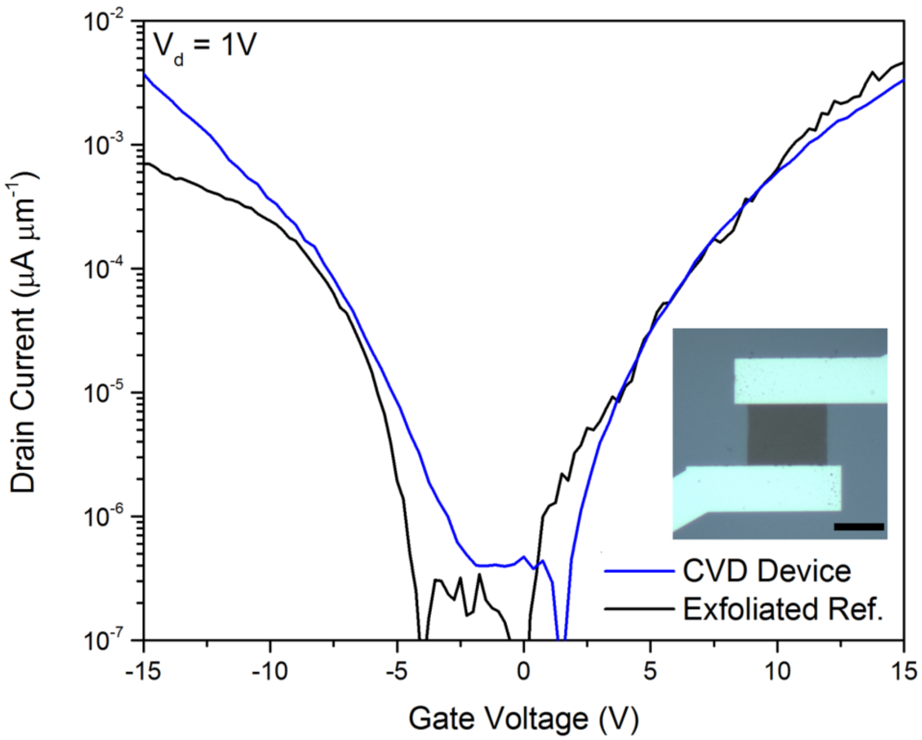

Supplementary Figure 13. Electrical performance of CVD WSe_2. I_d-V_g measurement of WSe_2 field-effect transistors fabricated from CVD grown and mechanically exfoliated WSe_2. Inset shows optical image of CVD WSe_2 device, scale bar is 8 μm.

Supplementary Tables

	AlN	Al_2O_3	$SrTiO_3$
***T* = 20°C**	-0.054	0.446	0.186
***T* = 875°C**	-0.057	0.445	0.188

Supplementary Table 1. Theoretical lattice misfit (f) calculated between WSe_2 and the various crystalline substrates investigated in this study.

Supplementary Notes

Supplementary Note 1: Frenkel-Kontorova Model to Explore Retention of Thermal-Expansion Mismatch Strains.

When viewed in the light of epitaxy, the thermal expansion mismatch strain observed in the samples is noteworthy, particularly in the case of the amorphous fused silica. With an amorphous substrate, one does not expect epitaxy, and hence one does not expect epitaxial strain. However, the amorphous substrate still represents a corrugated potential, albeit without long-range crystalline order, for the deposited film. It is interesting, then, to consider the amplitude of the corrugation necessary to sustain the thermal-expansion mismatch strain within the films. In order to explore the amplitude of the surface corrugation required to retain the strain arising due to thermal expansion mismatch, we explored a simple 2D Frenkel-Kontorova model[16]. Though the model is simple, it provides substantial insight into the considered problem, and enables exploration of the large systems. Though the model is best suited for epitaxy, it does allow exploration of the relationship between the corrugation of the potential and the retention of thermal mismatch strain.

The 2D Frenkel-Kontorova model considered here consists of a single plane of atoms connected by springs interacting with a substrate potential that has the same triangular symmetry (Supplementary Figure 1 (a)). One can think of these atoms as representing the plane of Se atoms in contact with the substrate. The springs strengths model the effective interactions between these atoms, that are, of course, determined by the layers W and Se atoms not indirect contact with the substrate. In dimensionless form, where all lengths are measured in terms of the substrate lattice parameter, a_s, and all energies measured in terms $\frac{1}{2}\kappa a_s^2$, with κ the spring constant of the model, the potential energy of the system can be written as:

$$E_{\text{potential}} = \sum_{i,j\in n.n.} \left(|\mathbf{r}_i - \mathbf{r}_j| - a_o\right)^2 + \sum_i V_o\left(6 - 2\left(\cos\left[\frac{4\pi y_i}{\sqrt{3}}\right] + \cos\left[\left(2\pi\left(3x_i - \sqrt{3}y_i\right)\right)/3\right] + \cos\left[\left(2\pi\left(3x_i + \sqrt{3}y_i\right)\right)/3\right]\right)\right)/8 \quad (1)$$

Here, $\mathbf{r}_i = x_i\mathbf{e}_1 + y_i\mathbf{e}_2$ is the (2D) vector describing the position of atom i ($\mathbf{e}_1$ and $\mathbf{e}_2$ are the unit vectors defining the coordinate system), V_o is the depth of the potential minima, a_o is the equilibrium spring length (measured in units of a_s), and the first sum is taken over nearest neighbors, counting each spring once.

The effects of thermal expansion mismatch are then explored as follows. A finite triangular shaped sample is defined. The initial configuration has each atom at a minimum of the substrate potential. A small random component is added to the positions of each atom, and the total potential energy of the system is then minimized. After minimization, the equilibrium length of the springs connecting the atoms, a_o, is reduced by a small amount (0.001 in dimensionless units), and the energy minimization is carried out again, using Mathematica's FindMinimum function with the initial positions chosen to be the positions found from the prior minimization. This process is repeated until the final value of $a_o = 0.99$.

In this model, the initial state corresponds to the growth temperature of the material. It is assumed that at the growth temperature, the materials are lattice matched. This is, in general, not true. However, for the case of the amorphous substrate, this is probably a reasonable assumption, as the growing film is not likely strained at the growth temperature. The reduction in the equilibrium spring length, then, is meant to model the thermal contraction of the WSe_2 film relative to the substrate. A reduction of 1% in the spring length is comparable to the thermal expansion mismatch between WSe_2 and SiO_2.

The model is then used to explore the amplitude of the substrate corrugation required to retain the thermal mismatch strain. The amplitude of the substrate potential, V_o, is reduced to the point where it can no longer sustain a 1% strain in the film. It is important to note that this strain

is sample size dependent, with larger samples requiring smaller, on average, corrugations to sustain the strain. Accordingly, we have considered an equilateral triangular sample containing 45,451 “atoms,” with an edge length of approximately 100 nm (this corresponds to minimizing a function of roughly 91,000 variables, exploration of larger sizes is computationally more difficult). We considered a range of potential energy corrugation amplitudes, V_o. For $V_\mathrm{o} << 1$, an example of the pattern of relaxation that we observe is shown in Supplementary Figure 1 (b). Here, the imposed thermal mismatch strain is 0.8% biaxial. The strain plotted is defined to be $\left(\frac{1}{2}\right)\left(\varepsilon_{xx} + \varepsilon_{yy}\right)$, with $\varepsilon_{ij} = \left(\frac{1}{2}\right)\left(\frac{\partial u_i}{\partial x_j} + \frac{\partial u_j}{\partial x_i}\right)$, and u_i the ith component of the displacement. The sample displays an interesting strain pattern. The interior of the sample retains much of the thermal mismatch strain, though the strain is not perfectly uniform. The edges of the sample show a clear strain relaxation. Through trial and error, we discovered that a potential corrugation amplitude of $V_\mathrm{o} \approx 5 \times 10^{-4}$ was sufficient to retain the substrate lattice parameter over most of the sample (the results are shown in Supplementary Figure 1 (b)). Recall that this amplitude is measured in units of $\frac{1}{2}\kappa a_s^2$. If one can compute the value of $\frac{1}{2}\kappa a_s^2$ for WSe_2, one can then assess the level of corrugation needed to sustain the thermal mismatch strain in the sample.

A value of $\frac{1}{2}\kappa a_s^2$ representing the properties of WSe_2 can be found as follows. Consider a spring network as shown in Supplementary Figure 1 (a). There are three springs per lattice point, and under a biaxial strain of -0.7% measured from their equilibrium length, they have a strain energy per unit cell of approximately:

$$E_{\mathrm{strain}} = 3\,\frac{1}{2}\kappa a_\mathrm{s}^2 (0.007)^2 \qquad (2)$$

This strain energy can be compared with the strain energy of the WSe_2 monolayer under equivalent strain.

To compute the strain energy of the WSe_2 monolayer, we employ linear elasticity theory. The lattice parameter and elastic constants for WSe_2 are computed as discussed below, arriving at the values $C_{11} = 120\ \mathrm{J\,m^{-2}}$ and $C_{12} = 23\ \mathrm{J\,m^{-2}}$, and $a_o = 3.361$ Å. Note that since the elastic constants represent the elastic properties of a 2D material, they have dimension of energy per area. Noting that the elastic energy of the film computed under a biaxial strain of ε is given by $(C_{11} + C_{12})\varepsilon^2$, and equating the two strain energies we find:

$$\frac{1}{3}(C_{11} + C_{12})\,A = \frac{1}{2}\kappa a_s^2 \tag{3}$$

with A being the unstrained area of a unit cell. Carrying out the computation, using the DFT lattice parameter, one concludes that for WSe_2, the parameter $\frac{1}{2}\kappa a_s^2 = 28.4$ eV. Noting that we can stabilize a strained film with a surface corrugation at the atomic scale of $V_o \approx 5 \times 10^{-4}$, we conclude that the film will be stable in the strained state if the corrugation at the atomic scale is of the order of 14 meV over the range of a single unit cell.

This required corrugation, then, sets a scale for the retention of thermal mismatch strain. It is much less than a typical covalent bond strength (of the order of 1 eV). Interestingly, this corrugation is very near to the strength of a typical van der Waals bond (of the order of 20-40 meV). We conclude that a covalent bond between the atoms of the growing film and that of the substrate is not necessary to enable strain tuning of the film via thermal expansion mismatch.

The elastic constants of a monolayer of WSe_2 were calculated using the plane-wave density functional theory (DFT) program VASP[1]. Projector augmented wave potentials were used for the ion-electron interactions,[2] and the PBE generalized gradient approximation was used for the exchange-correlation functional[3]. The system modeled was a WSe_2 monolayer, which was represented by a 3 atom cell with a 15.44 Å vacuum layer, normal to the monolayer. The plane wave energy cutoff was 600 eV, and the tetrahedron smearing method was used. The electronic

self-consistent loop's convergence criterion was set to 1 x 10^{-8}, and the system was relaxed until the Hellmann-Feynman forces on each atom were below 0.001 eV $Å^{-1}$. A gamma centered 29 × 29 × 1 Monkhorst pack grid was used to sample k-space. First, the system was relaxed allowing the cell and atomic positions to change. The lattice parameter for monolayer WSe_2 was found to be 3.316 Å. Using the relaxed system, the elastic constants were found using the method presented by de Jong *et. al*[4]. The 2D elastic constants for monolayer WSe_2 were calculated to be C_{11} = 120 J m^{-2} and C_{12} = 23 J m^{-2}.

Supplementary Note 2: Expected strain from lattice misfit.

Due to the fact that the WSe_2 is not covalently bound to the growth substrate we would not expect the strain to be transferred from the mismatch between the lattice constant of the substrate and the 2D material. However, to verify this we calculated the expected strain due to lattice misfit. The lattice misfit equation for heteroepitaxial strain is given by[5]:

$$f = \frac{a_{\mathrm{Sub}}(T) - a_{\mathrm{2D}}(T)}{a_{\mathrm{2D}}(T)} \quad (4)$$

where a_{Sub}, a_{2D}, and T are the lattice parameter of the substrate, the lattice parameter of the 2D material, and temperature respectively. In the case where $f > 0$ the film is expected to be under tensile strain, while for the case where $f < 0$ the film is expected to be under compressive strain. In our case we compute the expected lattice misfit both at room temperature as well as the growth temperature. The results are summarized in Supplementary Table 1, and indicate that we would expect that samples grown on AlN are under compressive strain while samples grown on Sapphire and STO are under tensile strain. This is highly inconsistent with the experimental results, which indicate that samples grown on AlN are under tensile strain, samples grown on STO are under compressive strain, and samples grown on Sapphire are relaxed, suggesting that the strain we observe in our samples does not originate from lattice mismatch.

Supplementary Methods

The furnace temperature profile as a function of the set point temperature for both zones was characterized to determine the actual sample temperature as well as the cross talk between furnace zones. This was used to adjust the set point of the upstream zone such that the selenium powder would reach the target temperature from excess heat provided by the downstream zone as well as to determine the actual temperature of the growth as compared to the furnace set point. A schematic of the furnace setup used for all growths performed in this study is shown in Supplementary Figure 7. For various substrates, several modifications were made to the general procedure used to grow monolayer samples on fused silica described in the main text. Optical images of WSe_2 grown on various substrates, as well as the dependence of growth morphology on Ar/H_2 ratio, growth time, and gas pressure are shown in Supplementary Figures 9, 10, 11, and 12 respectively. Additionally, photoluminescence imaging and atomic force microscopy were performed to verify sample uniformity and is shown in Supplementary Figure 3.

Growth on AlN: NaBr is employed as a promotor for the growth of WSe_2 instead of KBr. NaBr is mixed with WO_3 at 1:2 ratio. All the other growth parameters are kept the same as the method described in the manuscript.

Growth on sapphire (Al_2O_3): NaCl or KBr is used as a promotor for the growth of WSe_2 on sapphire. The promotor is mixed with WO_3 at the ratio of 1:2. All the other growth parameters are kept the same as the method described in the manuscript.

Growth on STO: NaBr is mixed with WO_3 at the ratio of 1:2. As the downstream furnace temperature reaches 875°C, the synthesis of the WSe_2 is initiated by introducing hydrogen. All the other growth parameters are kept the same as the method described in the manuscript.

Layer control: We found that the temperature of the WO_3/promoter boat plays a significant role in optimization of the WSe_2 thickness. By varying the temperature of the WO_3/promoter boat while keeping the other growth parameters constant, we could control the thickness of the material being grown. Synthesis initiated at 875°C results in predominantly monolayers domains. When the synthesis begins at 900°C, both WSe_2 monolayer and bilayer are observed. Growth initiated at temperatures greater than 900°C result in multilayer growth. This behavior can be attributed to increasing tungsten vapor pressure at high temperatures. It is important to note that for WSe_2 grown at different temperatures (Fig. 3 (c) and Supplementary Fig. 5), the WO_3/promoter boat and the target substrates were placed in separate furnace zones (shown schematically in Supplementary Fig. 7 (b)).

Device fabrication: Back-gated devices were fabricated on WSe_2 samples grown on fused silica and transferred to Si/SiO_2 (50 nm thick oxide) substrates as well as WSe_2 directly exfoliated on Si/SiO_2 (50 nm thick oxide) substrates to further verify material quality. All patterning was performed using electron beam lithography with PMMA C4 as the resist. Samples were first etched using XeF_2, and subsequently 40 nm thick Ni was deposited by thermal evaporation as the contact electrode. Both CVD and exfoliated WSe_2 devices show ambipolar behavior with similar on-currents as shown in Supplementary Figure 13.

Supplementary References

1. Kresse, G. & Hafner, J. Ab initio molecular dynamics for liquid metals. *Phys. Rev. B* **47,** 558–561 (1993).
2. Kresse, G., Joubert, D. From ultrasoft pseudopotentials to the projector augmented - wave method. *Phys. Rev. B* **59,** 1758 (1999).
3. Perdew, J. P., Ernzerhof, M., Burke, K. Generalized gradient approximation made simple. *Phys. Rev. Lett.* **77,** 3865–3868 (1996).
4. de Jong, M., Olmsted, D. L., van de Walle, A., Asta, M. First-principles study of the structural and elastic properties of rhenium-based transition-metal alloys. *Phys. Rev. B* **86,** 224101 (2012).
5. Ayers, J. E. *Heteroepitaxy of Semiconductors*. CRC Press, Boca Raton, FL (2007).
